# Feasibility of the Bag-Mediated Filtration System for Environmental Surveillance of Poliovirus in Kenya

**DOI:** 10.1007/s12560-019-09412-1

**Published:** 2019-11-02

**Authors:** Nicolette A. Zhou, Christine S. Fagnant-Sperati, Evans Komen, Benlick Mwangi, Johnstone Mukubi, James Nyangao, Joanne Hassan, Agnes Chepkurui, Caroline Maina, Walda B. van Zyl, Peter N. Matsapola, Marianne Wolfaardt, Fhatuwani B. Ngwana, Stacey Jeffries-Miles, Angela Coulliette-Salmond, Silvia Peñaranda, Jeffry H. Shirai, Alexandra L. Kossik, Nicola K. Beck, Robyn Wilmouth, David S. Boyle, Cara C. Burns, Maureen B. Taylor, Peter Borus, John Scott Meschke

**Affiliations:** 1 Department of Environmental and Occupational Health Sciences, University of Washington, 4225 Roosevelt Way NE, Suite 100, Seattle, WA 98105 USA; 2 Centre for Viral Research, Kenya Medical Research Institute, Mbagathi Road, P.O. Box 54628, Nairobi, 00200 Kenya; 3 Kenya Ministry of Health, Afya House, Cathedral Road, P.O. Box 30016, Nairobi, 00100 Kenya; 4 Department of Medical Virology, Faculty of Health Sciences, University of Pretoria, Private Bag X323, Arcadia, 0007 South Africa; 5 IHRC, Inc. (contracting agency to the Division of Viral Diseases, Centers for Diseases Control and Prevention, Atlanta, GA 30329, USA), 2 Ravinia Drive, Suite 1200, Atlanta, GA 30329 USA; 6 Division of Viral Diseases, Centers for Disease Control and Prevention, 1600 Clifton Road NE, Mailstop H17-6, Atlanta, GA 30329 USA; 7 PATH, 2201 Westlake Ave, Suite 200, Seattle, WA 98121 USA

**Keywords:** BMFS, Wastewater, Environmental monitoring, Environmental surveillance, Poliovirus, Two-phase separation

## Abstract

The bag-mediated filtration system (BMFS) was developed to facilitate poliovirus (PV) environmental surveillance, a supplement to acute flaccid paralysis surveillance in PV eradication efforts. From April to September 2015, environmental samples were collected from four sites in Nairobi, Kenya, and processed using two collection/concentration methodologies: BMFS (> 3 L filtered) and grab sample (1 L collected; 0.5 L concentrated) with two-phase separation. BMFS and two-phase samples were analyzed for PV by the standard World Health Organization poliovirus isolation algorithm followed by intratypic differentiation. BMFS samples were also analyzed by a cell culture independent real-time reverse transcription polymerase chain reaction (rRT-PCR) and an alternative cell culture method (integrated cell culture-rRT-PCR with PLC/PRF/5, L20B, and BGM cell lines). Sabin polioviruses were detected in a majority of samples using BMFS (37/42) and two-phase separation (32/42). There was statistically more frequent detection of Sabin-like PV type 3 in samples concentrated with BMFS (22/42) than by two-phase separation (14/42, *p* = 0.035), possibly due to greater effective volume assayed (870 mL vs. 150 mL). Despite this effective volume assayed, there was no statistical difference in Sabin-like PV type 1 and Sabin-like PV type 2 detection between these methods (9/42 vs. 8/42, *p* = 0.80 and 27/42 vs. 32/42, *p* = 0.18, respectively). This study demonstrated that BMFS can be used for PV environmental surveillance and established a feasible study design for future research.

**Electronic supplementary material:**

The online version of this article (10.1007/s12560-019-09412-1) contains supplementary material, which is available to authorized users.

## Introduction

Environmental sampling of wastewater and wastewater-impacted surface waters analyzed for poliovirus (PV), as a supplement to acute flaccid paralysis (AFP) surveillance, plays an important role in the detection of wild PV (WPV) and vaccine-derived PV (VDPV) transmission (Asghar et al. 2014; Cowger et al. 2017; Hovi et al. 2012). Environmental surveillance can assist in determining where PV is circulating in locations where AFP surveillance fails to detect poliovirus paralytic cases and whether performance is substandard or meeting indicators (World Health Organization [WHO] 2015). Environmental surveillance has also demonstrated the elimination of WPV in Egypt (El Bassioni et al. 2003; Hovi et al. 2005) and India (Chowdhary and Dhole 2008; Deshpande et al. 2003; Shukla et al. 2013) in support of AFP surveillance data, and the resurgence of WPV circulation in previously documented polio-free areas (Manor et al. 1999, 2007; Anis et al. 2013). The disappearance of the PV type 2 Sabin vaccine strain after its removal from the live attenuated oral poliovirus vaccine (OPV) in April 2016 has been monitored via environmental surveillance and this will be useful in monitoring the disappearance of all PV vaccine strains after cessation of OPV (Diop et al. 2017; Hovi et al. 2012; Lopalco 2017; WHO 2013). Therefore, the WHO has expanded environmental surveillance during the past 5 years (Gardner et al. 2018; Maes et al. 2017; WHO 2015).

Routine environmental surveillance of PV began in 1984 (WHO 2015). The standard WHO-supported concentration method (two-phase separation) involves collecting a 1-L grab sample, followed by reverse cold-chain sample transport to the laboratory for processing (WHO 2003, 2015). Samples are processed by an overnight incubation with the addition of polyethylene glycol (PEG) and dextran. This method has been used in Kenya for PV environmental surveillance since October 2013, initially for three sites in Nairobi and expanding to five sites in 2014 (Borus et al. 2015). Routine PV environmental surveillance has been increasing globally using the two-phase separation method, with 38 countries participating in 2017 (Gardner et al. 2018; Maes et al. 2017; Snider et al. 2016).

To facilitate PV environmental surveillance, a novel, alternative method for sample collection and processing was developed, the bag-mediated filtration system (BMFS) (Fagnant et al. 2014, 2018). BMFS enables collection of large sample volumes (3–6 L wastewater or wastewater-impacted waters) and the ability for in-field filtration through a charged cartridge filter. Prior to field sampling, bacteriophage MS2 can be pre-seeded onto the filters as an internal control (Fagnant et al. 2017). After filtration, the samples are transported by reverse cold chain to the laboratory for further processing. In the laboratory, a preservative mixture is added to improve the survival of PV over time on the filters followed by filter elution and secondary concentration (Fagnant et al. 2017, 2018; Zhou et al. 2018). Processing large volumes allows for an increased effective volume assayed, while on-site filtration eliminates transport of large water samples and reduces required laboratory space.

The objectives of this study were to (1) test the feasibility and applicability of the BMFS for monitoring PV in environmental samples (2) establish a study design and logistics for use in a future BMFS validation study, and (3) analyze PV by three methods. These methods included the standard method used for PV detection and two versions of a method developed for enterovirus (EV) detection. This study examined their applicability for PV detection. Environmental samples were collected from four Kenyan sites, processed using the BMFS and two-phase separation methods and analyzed for PV.

## Materials and Methods

### Training

Technicians from the Kenya Medical Research Institute (KEMRI) Center for Virus Research in Nairobi, Kenya and the University of Pretoria in Pretoria, South Africa were trained in-person on the use of the BMFS field kit and laboratory protocols (Fagnant et al. 2014, 2017, 2018). Trainees received detailed illustrated protocols, a one-page “quick-sheet” reference protocol, and a training video describing the BMFS field sampling protocol. Training on a supplemental “bucket” modification was completed by telephone with a detailed illustrated protocol. This modification was developed to safely transport large water samples on cold chain for sites with safety or security concerns (Zhou et al. 2018).

### Study Design

**Fig. 1:**
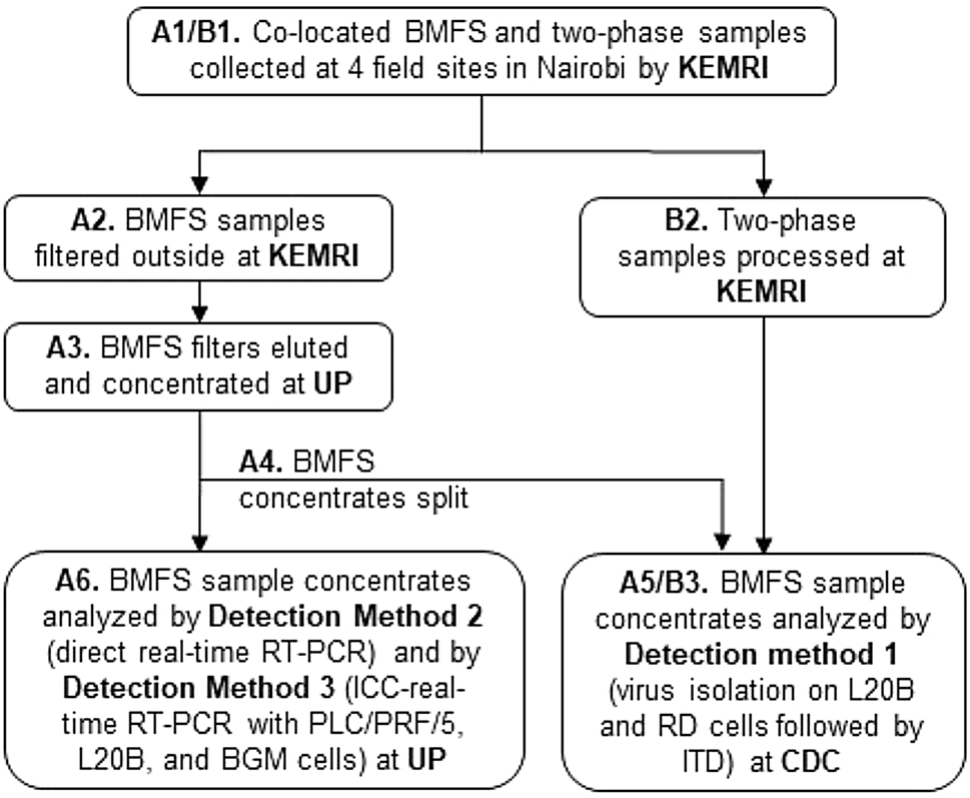
Workflow of collected environmental samples. Bag-mediated filtration system (BMFS) samples were collected concurrently with 1 L grab samples for two-phase concentration according to World Health Organization protocols. *KEMRI* Kenya Medical Research Institute, *UP* University of Pretoria, *CDC* US Centers for Disease Control and Prevention, *ITD* intratypic differentiation

Environmental samples were collected from April 14, 2015 to September 7, 2015 at four sites in Nairobi, Kenya, twice per month, and analyzed for PV (Fig. 1). Samples were obtained from the Motoine River bordering the Kibera informal settlement (Kibera), a latrine waste stream that connects to the Mathare River (Starehe), and two sewer conveyance lines in the Eastleigh neighborhood (Eastleigh A and B). In 2013 and 2014, these sites were chosen for the WHO environmental surveillance program based on discussions among KEMRI, the Kenya Ministry of Health, and WHO. Sampling was conducted by a field staff team made up of two KEMRI field technicians and a supervisor. At some sites, a water utility staff member and/or community health worker were present. BMFS sampling frequency was set to coincide with routine WHO two-phase sampling activities, and occurred over 12 sampling days (Online Resource, Table S4). Environmental samples processed by the two-phase separation method were collected first, and a second sample was collected for the BMFS. Samples were collected within 5 min and a 1-m radius of each other. Samples were collected in the late morning/early afternoon, with sampling beginning between 10:30 and 14:15 at the Kibera site (*n* = 15), 09:00–12:15 at the Starehe site (*n* = 15), 09:50–11:15 at the Eastleigh A site (*n* = 13), and 09:45–11:45 at the Eastleigh B site (*n* = 13). During the first 14 sampling events of the study period (April to May 2015), two BMFS samples were collected sequentially (within 5 min) at the same site to determine variability in BMFS sampling: four each at Kibera and Starehe, and three each at Eastleigh A and Eastleigh B (Table 1).

**Table 1:** Detection of SL1, SL2, and SL3 in sequentially collected BMFS samples as measured by WHO algorithm

Date (dd/mm/yyyy)	Site	SL1			SL2			SL3		
		BMFS samples		Concordant positive method	BMFS samples		Concordant positive method	BMFS samples		Concordant positive method
		Rep 1	Rep 2		Rep 1	Rep 2		Rep 1	Rep 2	
14/04/2015	Kibera	+	−	−	+	+	+	+	+	+
14/04/2015	Starehe	−	−	−	+	+	+	+	+	+
28/04/2015	Starehe	−	−	−	−	+	−	+	+	+
28/04/2015	Eastleigh A	+	+	+	+	−	−	−	−	−
28/04/2015	Eastleigh B	−	−	−	−	−	−	+	+	+
04/05/2015	Kibera	+	+	+	+	+	+	+	+	+
13/05/2015	Kibera	−	−	−	−	−	−	+	+	+
13/05/2015	Starehe	+	−	−	−	+	−	+	−	−
13/05/2015	Eastleigh A	+	−	−	+	+	+	+	−	−
13/05/2015	Eastleigh B	−	−	−	+	+	+	−	−	−
26/05/2015	Kibera	−	+	−	+	+	+	+	+	+
26/05/2015	Starehe	−	−	−	+	+	+	+	+	+
26/05/2015	Eastleigh A	−	−	−	+	+	+	−	−	−
26/05/2015	Eastleigh B	+	−	−	+	+	+	+	+	+

*SL1* Sabin-like poliovirus type 1, *SL2* Sabin-like poliovirus type 2, *SL3* Sabin-like poliovirus type 3, *BMFS* bag-mediated filtration system, *WHO (World Health Organization) algorithm* virus isolation on L20B and RD (human rhabdomyosarcoma) cells followed by ITD (intratypic differentiation), *Concordant positive method* sequentially collected BMFS sample results combined by considering discordant poliovirus results to be negative and by considering concordant poliovirus results as a single sample

### Study Permissions

This study was approved by KEMRI, the National Commission for Science, Technology, and Innovation (NACOSTI), and the Research Ethics Committee, Faculty of Health Sciences, University of Pretoria Ethics Reference no: 119/2017. Appropriate import and export permits were obtained from the Department of Health South Africa and the United States Centers for Disease Control and Prevention (CDC) to facilitate intercountry transfer of samples and derivatives.

### BMFS Samples

#### MS2 Seeding and Analysis

Prior to sample filtration, disposable encapsulated 2-inch ViroCap filters (Scientific Methods, Inc., Granger, IN, USA) were seeded with a known titer of bacteriophage MS2. Briefly, a stock of MS2 (ATCC 15,597-B1) was prepared at the University of Washington by confluent lysis of *Escherichia coli* F-amp (ATCC 700,891) using the double agar layer method (Adams 1959; US EPA 2000) followed by extraction using Vertrel XF (DuPont, Wilmington, DE, USA) (Mendez et al. 2000). The MS2 and *E. coli* F-amp stocks were transported to KEMRI and the University of Pretoria on cold packs. A double agar layer assay was conducted upon arrival at each location to assess titer of the transported stocks, and the MS2 stock was then diluted at KEMRI to a concentration of 10^5^ plaque forming units (PFU)/mL. To minimize loss of virus viability due to multiple freeze-thaws, the diluted MS2 stock was stored in 1-mL aliquots at − 80 °C. To seed the filters, one aliquot was thawed and the full volume was added to 175 mL 1 × phosphate-buffered saline (PBS), pH 7.4 (Fisher Scientific International, Inc., Hampton, NH, USA). The spiked PBS was poured into the ViroCap filter inlet, held for 20 min, and then pumped through the filter outlet at a rate of ~ 85 mL/min using a peristaltic pump (Control Company, Webster, TX, USA). Filters were pre-seeded at KEMRI within 24 h of sample filtration and stored at 4 °C prior to use (stability data provided in the Online Resource).

Infectious MS2 was enumerated from the filter eluate at the University of Pretoria by the double agar layer method on *E. coli* F-amp host (Adams 1959; US EPA 2000) to determine the applicability of including this step in the BMFS protocol. Briefly, 100 µL aliquots of sample dilutions in 1 × PBS, pH 7.4, were combined with 100 µL *E. coli* F-amp (log-phase growth) in 6–7 mL molten bacto agar, then poured onto 100-mm tryptic soy agar petri plates. Plates were incubated (37 °C, 18–20 h) and plaques were counted. Each sample dilution was plated in duplicate.

#### Sample Collection and Processing

BMFS samples were collected and filtered as previously described (Fagnant et al. 2018). Briefly, samples were collected in the 6-L sampling bag with a pre-screen mesh (249-µm pore size) over the opening; the bag was hung on a tripod, an MS2 pre-seeded ViroCap filter was attached to the outlet port of the bag, and the sample was filtered by gravity. The filter was transported on cold packs to the KEMRI laboratory. Beginning in May 2015, filtration was completed at a manhole over a sewer conveyance line on the KEMRI campus rather than at the field site, with the implementation of the bucket modification as described elsewhere (Zhou et al. 2018). Briefly, sample transport with the bucket modification included cold chain and water-tight secondary containment via a 5-gallon bucket with a water-tight lid. After sample collection (Fig. 1, A1), samples were transported to KEMRI, stored at 4 °C, and filtered outside by gravity within 24 h of receipt (Fig. 1, A2). Filtrate drained into a sewer. Total collection time at the four environmental surveillance sites was recorded to determine the feasibility of utilizing this study design for future environmental surveillance work. Volume filtered was measured to enable calculation of the effective volume assayed, and to determine the typical volume of Kenyan wastewater able to be processed prior to filter clogging. In the laboratory, a preservative mixture [175 mL; 2% sodium benzoate (Alfa Aesar, Ward Hill, MA, USA) and 0.2% calcium propionate (TCI America, Portland, OR, USA)] was poured into the filter inlet after sample filtration, held for 20 min, and then pumped through the filter outlet using a peristaltic pump.

**Fig. 2:**
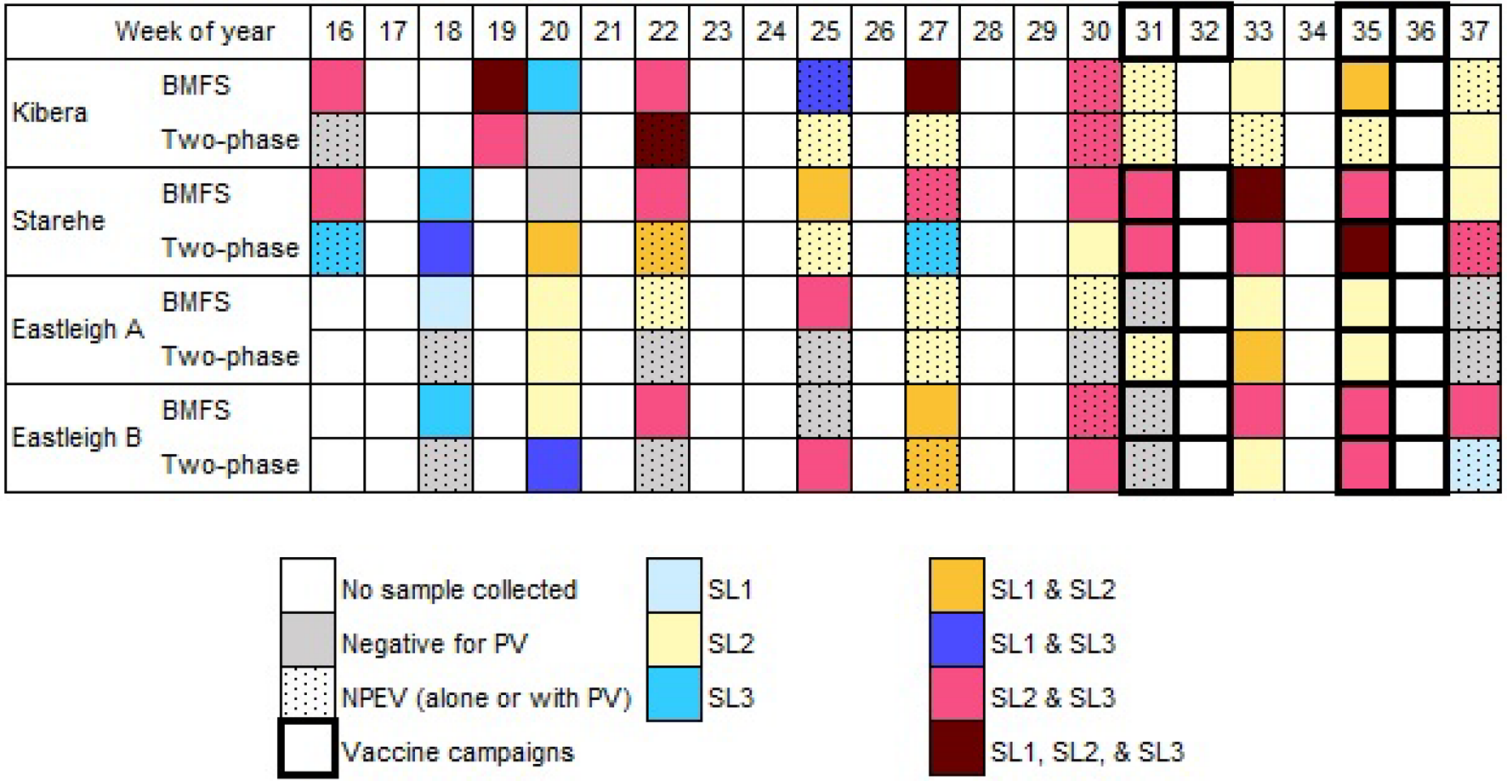
Detection of Sabin-like polioviruses during April–September 2015 in bag-mediated filtration system (BMFS) and two-phase samples from four study sites in Nairobi, Kenya. *BMFS* bag-mediated filtration system, *PV* poliovirus, *NPEV* non-polio enterovirus, *SL1* Sabin-like poliovirus type 1, *SL2* Sabin-like poliovirus type 2, *SL3* Sabin-like poliovirus type 3

Filters were shipped to the University of Pretoria in insulated boxes with ice packs for virus elution and secondary concentration as previously described (Fagnant et al. 2018) (Fig. 1, A3). The ViroCap filters were eluted with a 1.5% beef extract (BBL™ Beef Extract powder; Becton, Dickinson and Company, Sparks, MD, USA) and 0.05 M glycine (Merck, Darmstadt, Germany) solution at pH 9.5 using a peristaltic pump at a rate of ~ 85 mL/min. Samples were stored at 4 °C for no longer than 5 days until secondary concentration using PEG 8000 (AMRESCO, Solon, OH, USA)/NaCl (Merck) precipitation, and resuspended in 10 mL 1 × PBS. Concentrated samples were divided (Fig. 1, A4), with 4 mL sent to the CDC for analysis, and 6 mL remaining at the University of Pretoria for chloroform extraction and analyses (Fig. 1, A5 and A6). Storage times at the sites and shipping times from the University of Pretoria to CDC were recorded to assist in determining bottlenecks in sample processing and if further logistical planning was required for future environmental surveillance studies utilizing this design.

#### PV Detection

BMFS samples were analyzed for SL1, SL2, SL3, WPV1, and WPV3 by three detection methods. (1) WHO algorithm: virus isolation and amplification using L20B recombinant murine cells and human rhabdomyosarcoma (RD) cells followed by ITD using real-time reverse transcription polymerase chain reaction (rRT-PCR) according to WHO protocols at the CDC; (2) direct RT-PCR: direct real-time RT-PCR at the University of Pretoria; and (3) ICC-RT-PCR: integrated cell culture (ICC)-real-time RT-PCR with PLC/PRF/5, L20B, and buffalo green monkey (BGM) cell lines at University of Pretoria.

##### WHO Algorithm: Virus Isolation on L20B and RD Cells Followed by ITD

BMFS samples were analyzed at the CDC according to the WHO algorithm (WHO 2015) and ITD for SL1, SL2, SL3, WPV1, WPV3, panPV, and panEV (Fig. 1, A5). Virus isolation on two cell lines (L20B and RD cells) was performed prior to detection of PV in the cell culture supernatant (virus isolate) by ITD using rRT-PCR (Poliovirus ITD 4.0/4.1 rRT-PCR Kit [CDC, Atlanta, GA, USA]) (WHO 2015). Primers and probes used for detection of SL1, SL2, and SL3 are shown in the Online Resource (Table S1) (Kilpatrick et al. 2009). Primers and probes used for detection of WPV1 and WPV3 were in the Poliovirus ITD 5.0 rRT-PCR Kit, as described in Gerloff et al. (2018). Real-time RT-PCR was performed using an Applied Biosystems® 7500 thermocycler (Applied Biosystems, Foster City, CA, USA), and the programs used are shown in the Online Resource (Table S2).

##### Direct RT-PCR: Direct Real-Time RT-PCR

For direct real-time RT-PCR conducted at the University of Pretoria (Fig. 1, A6), the nucleic acid was extracted from the chloroform-extracted BMFS secondary concentrate using the NucliSENS® easyMAG® instrument and accessory products (bioMérieux SA, Marcy-l’Étoile, France) according to the manufacturer’s instructions, with a 1000-µL input volume and nucleic acid elution in 100 µL.

For WPV1 and WPV3 detection, the standard WHO Poliovirus ITD 4.0/4.1 rRT-PCR Kit was used as described in the ITD 5.0 rRT-PCR Kit (Gerloff et al. 2018). All assays were analyzed with QuantStudio™ 5 Real-Time PCR System (Applied Biosystems). The QuantStudio has not been validated for the ITD suite of assays and results may not be representative of standard GPLN results.

The standard WHO Poliovirus ITD Kit was not used for SL1, SL2, or SL3 detection at the University of Pretoria. Instead, primers and probes from Nijst et al. (2013) were synthesized by Applied Biosystems (Online Resource, Table S1), and real-time RT-PCR analysis was performed using a Lightcycler 2.0 (Roche Diagnostics, Mannheim, Germany) and QuantiTect® Probe RT-PCR Kit (Qiagen, Hilden, Germany). A 5-µL input volume of the nucleic acid extract was used. The RT-PCR programs used are shown in the Online Resource (Table S2). Nuclease-free water (Promega Corp., Madison, WI, USA) was used as the negative control.

##### ICC-RT-PCR: ICC-Real-Time RT-PCR with PLC/PRF/5, L20B, and BGM Cell Lines

Cell culture virus amplification was performed prior to detection using real-time RT-PCR at the University of Pretoria (Fig. 1, A6) (Grabow et al. 1999). Three cell lines used were PLC/PRF/5 human hepatoma cell line, L20B recombinant murine cell line, and BGM cell line (Online Resource, Table S3). Cells were propagated at 37 °C and 5% CO_2_ in MEM growth medium (Online Resource, Table S3). Prior to infection, 25 cm^2^ (T-25) flasks were seeded with 4 mL of 10^5^ cells/mL and incubated at 37 °C and 5% CO_2_ for 36–48 h.

The chloroform-extracted BMFS secondary concentrate (3.5 mL) was treated with 100 µL penicillin–streptomycin-neomycin solution (penicillin 5000 U/mL, streptomycin 5 mg/mL, neomycin 10 mg/mL) (Sigma-Aldrich Co., St. Louis, MO, USA) and 100 µL nystatin suspension (10,000 U/mL) (Sigma-Aldrich Co.) at room temperature (~ 25 °C) for 1 h. For infection, two T-25 flasks of each cell line were used. Confluent monolayers were washed with 1 mL serum-free medium (held at ~ 25 °C, 1–1.5 h). The wash medium was aspirated and 0.5-mL treated, chloroform-extracted BMFS secondary concentrate was immediately inoculated onto each flask and allowed to adsorb at room temperature (~25 °C) for 1 h. After adsorption, 4 mL of the appropriate maintenance medium was added to each flask (Online Resource, Table S3). The flasks were incubated (37 °C, 5% CO_2_) and monitored daily (up to 7 days post infection) for cytopathic effect (CPE). An uninfected T-25 flask of each cell type was included in all steps as a negative control. When CPE (> 50%) was recorded, the flasks were stored at 4 °C until harvesting (7 days post infection). The infected cell cultures were harvested by scraping the cell monolayer off the surface of the flask into the surrounding medium. An equal volume of harvested cell culture from the two flasks of each cell type was mixed thoroughly and used for further molecular analysis for enteroviruses, including PV, as well as for blind passage onto monolayers of Vero African green monkey cell line (ECACC 84,113,001) on narrow coverslips inserted into cell culture tubes (Yadav and Tyagi 2008). The infected Vero cell cultures were incubated at 37 °C on a roller apparatus and examined daily for CPE. Once CPE was observed, the coverslips were stained with hematoxylin and eosin and examined for virus-specific inclusion bodies as previously described (Malherbe and Strickland-Cholmley 1980). For molecular analysis, the harvested cell cultures were freeze-thawed three times and the nucleic acid was extracted using the NucliSENS easyMAG instrument and accessory products (bioMérieux SA) according to the manufacturer’s instructions, with a 100-µL input volume and nucleic acid elution in 50 µL. Real-time RT-PCR analysis was performed as described above in “Direct RT-PCR” section.

### Two-Phase Samples

Two-phase samples were collected according to WHO protocols (WHO 2015). One-liter grab samples were collected and transported on cold packs to KEMRI (Fig. 1, B1). Aliquots of 500 mL per sample were concentrated at KEMRI using the two-phase separation PEG/dextran method as previously described (Fig. 1, B2) (WHO 2015). Briefly, samples were centrifuged and the pellet was saved to add at a later step. PEG 6000 (Sigma-Aldrich, St. Louis, MO, USA), dextran (Pharmacosmos, Holbaek, Denmark), and NaCl (Sigma-Aldrich) were added to the supernatant, mixed for one hour, added to a separatory funnel, and held overnight at 4 °C. The lower phase and interphase in the separation funnel were collected, combined with the pellet, and extracted using chloroform. The upper aqueous phase from the chloroform extraction was collected (approximately 10 mL; 7–12 mL), and sample concentrates were sent to the CDC in Atlanta, GA, USA. Two-phase samples were analyzed at the CDC according to the WHO virus isolation algorithm (WHO 2015) followed by intratypic differentiation (ITD) for SL PV type 1 (SL1), SL PV type 2 (SL2), SL PV type 3 (SL3), WPV type 1 (WPV1), WPV type 3 (WPV3), panPV, and panEV (Fig. 1, B3) (WHO algorithm, see above “WHO Algorithm” section). This algorithm, combined with VP1 sequencing, can distinguish VDPV from SL PV and WPV (Gerloff et al. 2018).

### Analyses

A generalized linear mixed model was performed to determine if there was a correlation between MS2 and PV detection. Sample site was treated as a non-random variable effect. The McNemar mid-p test was used to determine the significance of the difference between samples (*i.e.*, BMFS and two-phase samples collected within 5 min and a 1-meter radius of each other, and individual BMFS samples measured by different detection methods) (Fagerland et al. 2013; McNemar 1947). The odds ratio (OR) and the 95% confidence intervals (CI) on the OR were also calculated. Results were considered significant with a mid-*p* value < 0.05. Analyses were conducted using MS Excel 2016 and R version 3.4.3.

Results from the two sequentially collected BMFS samples (BMFS-1 and BMFS-2; April to May 2015) were analyzed by three methods. First, they were treated as a single composite sample result (*n* = 14) by the (1) concordant positive method (Table 1). Additionally (2) BMFS-1 (*n* = 14) and single BMFS samples (*n* = 28) and (3) BMFS-2 (*n* = 14) and single BMFS samples (*n* = 28) were combined into two separate sample sets. The concordant positive method considered discordant PV results to be negative and concordant PV results as a single sample, as to not bias the comparison between the BMFS and two-phase methods. Single BMFS samples were collected from June to September 2015 (*n* = 28), for a total of 42 BMFS samples analyzed. Single two-phase samples were collected throughout the study period (*n* = 42).

## Results

### BMFS Sample Logistics

This study established a study design and logistics required to conduct a future environmental surveillance study using the BMFS by identifying the scheme logistics, shipping logistics, and processing timeframes needed. An average of 2.9 ± 0.1 L (95% CI; *n* = 54) was filtered using the BMFS, and filtration took an average of 70.1 ± 3.9 min (95% CI; *n* = 55). Use of the bucket modification at two sites due to safety and security concerns enabled continued collection of BMFS samples alongside two-phase samples. This modification was a preferred strategy by the field staff as it enabled concurrent filtration of all samples outside near KEMRI; therefore, it was implemented at all sites during the last 9 sampling days (Online Resource, Table S4). During these 9 sampling days, overall sample collection (from the beginning of the first sample collection to arrival back at KEMRI) took an average of 4.7 h (*n* = 9) (Online Resource, Table S4).

Protocols were instituted to inform on and enhance sample integrity, including the addition of MS2 and preservatives, timely shipping, and cold-chain techniques. MS2 was seeded in all filters prior to sample filtration (*n* = 56), and MS2 recovery efficiency ranged from no detection to 500%, with a median recovery value of 8.8%. No correlation existed between MS2 recovery and positive or negative PV detection in BMFS (*p* = 0.81, 0.27, and 0.54 for SL1, SL2, and SL3, respectively) and two-phase (*p* = 0.23, 0.11, and 0.62 for SL1, SL2, and SL3, respectively) samples when measured using the WHO algorithm. After filtration, filters were stored (4 °C) and preservatives were added prior to shipping of the filters to the University of Pretoria for processing (*n* = 56; Online Resource, Table S4). After elution and secondary concentration at the University of Pretoria, portions of the BMFS sample concentrates were stored prior to shipment to the CDC (− 80 °C, 4.5–9.5 months). Samples were shipped to the CDC in two batches of 30 samples each with dry ice replenished throughout (shipping time 14 and 17 days, respectively).

Samples received at the CDC were stored (− 20 °C or − 80 °C, 1 week to 3.5 months) before processing using the WHO algorithm. For BMFS samples at the University of Pretoria measured by direct RT-PCR, 1-mL aliquots of chloroform-extracted secondary concentrates were frozen (− 20 °C) prior to nucleic acid extraction. The nucleic acids were stored until real-time RT-PCR was completed (− 80 °C, < 1 month). For samples at the University of Pretoria measured by ICC-RT-PCR, cell culture was initiated within 9 days after secondary concentration for 86% of samples, and the chloroform-extracted secondary concentrates were stored at 4 °C until inoculation into cell lines. For the other 14% of samples, the secondary concentrates were stored (− 20 °C, 20 days) prior to inoculation of the cell lines due to the large quantity of samples received.

### BMFS Samples

No statistical difference in PV detection was noted between the two sequentially collected BMFS samples (BMFS-1 and BMFS-2) for all three detection methods used (WHO algorithm, direct RT-PCR, and ICC-RT-PCR: *p* = 0.22, 1, and 0.5 for SL1, *p* = 0.63, 0.5, and 0.63 for SL2, and *p* = 0.25, 0.13, and 0.25 for SL3; *n* = 14). Combining sequentially collected BMFS samples (*n* = 14; April to May 2015) and single BMFS samples (*n* = 28; June to September 2015) results in a total of 42 samples. When detected by the WHO algorithm and analyzed using the concordant positive method (Table 1), SL1, SL2, and SL3 were detected in 19.0%, 76.2%, and 52.4% of BMFS samples, respectively (Table 2; *n* = 42). When considering the BMFS-1 and single BMFS sample dataset, SL1, SL2, and SL3 were detected in 28.6%, 78.6%, and 57.1% of BMFS samples, respectively (*n* = 42). When considering the BMFS-2 and single BMFS sample dataset, SL1, SL2, and SL3 were detected in 21.4%, 81.0%, and 52.4% of BMFS samples, respectively (*n* = 42). The following analyses (comparing BMFS samples analyzed by different detection methods, and comparing between the BMFS and two-phase results) utilized the concordant positive method.

**Table 2:** PV detection in 1-L grab samples with 500-mL processed by two-phase concentration and measured by the WHO algorithm versus BMFS samples by three different detection methods

Collection/detection method	SL1 (%)	SL2 (%)	SL3 (%)	*n*
Two-phase				
(1) Virus isolation on L20B and RD cells followed by ITD (CDC)	21.4	64.3	33.3	42
BMFS				
(1) Virus isolation on L20B and RD cells followed by ITD (CDC)	19.0	76.2	52.4	42
(2) Direct real-time RT-PCR (UP)	0.0	14.3	31.0	42
(3) Integrated cell culture real-time RT-PCR with PLC/PRF/5, L20B, and BGM cell lines (UP)	31.0	81.0	66.7	42
After amplification on PLC/PRF/5 cells	16.7	61.9	47.6	42
After amplification on L20B cells	23.8	69.0	45.2	42
After amplification on BGM cells	2.4	31.0	16.7	42

*PV* poliovirus, *BMFS* bag-mediated filtration system, *WHO* World Health Organization, *SL1* Sabin-like PV type 1, *SL2* Sabin-like PV type 2, *SL3* Sabin-like PV type 3, *RD* human rhabdomyosarcoma, *ITD* intratypic differentiation, *CDC* Centers for Disease Control and Prevention, *RT* reverse transcription, *PCR* polymerase chain reaction, *UP* University of Pretoria, *BGM* buffalo green monkey

The rate of PV detection using the three different detection methods varied (Table 2 and Online Resource, Table S6). PV detection in BMFS samples was not significantly different when measured by the WHO algorithm compared to ICC-RT-PCR (*p* = 0.092, 0.125, and 0.092 for SL1, SL2, and SL3, respectively; Online Resource, Table S6). PV detection in BMFS samples was statistically less frequent by direct RT-PCR compared to WHO algorithm (*p* = 3.9 × 10^−3^, 1.49 × 10^−8^, and 6.3 × 10^−3^ for SL1, SL2, and SL3, respectively) and ICC-RT-PCR (*p* = 1.2 × 10^−4^, 3.73 × 10^−9^, and 3.05 × 10^−5^ for SL1, SL2, and SL3, respectively; Online Resource, Table S6). Discordant results favored the WHO algorithm when compared to direct RT-PCR, in all cases except with one SL3. Discordant results favored ICC-RT-PCR when compared to direct RT-PCR in all cases. The most frequently detected PV also varied with the detection method. SL2 was the most frequently detected PV, followed by SL3, and SL1, when measured using the WHO algorithm and ICC-RT-PCR (Table 2). In contrast, SL3 was the most frequently detected PV when measured using direct RT-PCR. PV detection also varied after amplification in the three cell lines in ICC-RT-PCR (Table 2 and Online Resource, Table S7). PV prevalence in BMFS samples was not statistically different between PLC/PRF/5 and L20B cells (*p* = 0.344, 0.388, and 0.774 for SL1, SL2, and SL3, respectively; Online Resource, Table S7). In contrast, PV was statistically detected less frequently after amplification in BGM cells than after amplification in L20B cells (*p* = 2.0 × 10^−3^, 7.63 × 10^−5^, and 2.4 × 10^−3^ for SL1, SL2, and SL3, respectively) or PLC/PRF/5 cells (*p* = 1.6 × 10^−2^, 5.2 × 10^−4^, and 2.6 × 10^−3^ for SL1, SL2, and SL3, respectively; Online Resource, Table S7).

### PV Detection Comparison in Matched BMFS and Two-Phase Samples Analyzed by the WHO Algorithm

The BMFS and two-phase samples were both concentrated to approximately 10 mL resulting in a 290- and 50-fold concentration, respectively (effective volume assayed of 870 mL per BMFS sample vs. 150 mL per two-phase sample after inoculation). Poliovirus was detected in a majority of samples using the standard WHO algorithm (Table 3) with at least one PV in 88.1% (37 of 42) and 76.2% (32 of 42) of BMFS and two-phase samples, respectively (Fig. 2). WPV1, WPV3, and VDPV were not detected in samples by any of the detection methods. There was statistically more frequent detection of SL3 in BMFS than in two-phase samples (52.4% [22 of 42] and 33.3% [14 of 42], respectively; *p* = 0.035), with an increased odds ratio of SL3 detection in BMFS samples compared to two-phase samples (OR 3.7 [1.0–13 95% CI]) (Table 3). For SL1, there was no statistical difference in the frequency of detection between BMFS and two-phase samples (19.0% [8 of 42] and 21.4% [9 of 42], respectively; *p* = 0.80) (Table 3). There was no statistical difference in the frequency of SL2 detection in BMFS and two-phase samples (76.2% [32 of 42] and 64.3% [27 of 42], respectively; *p* = 0.18) (Table 3). SL2 was the most frequently detected PV in both BMFS and two-phase samples, followed by SL3, and SL1 (Table 3).

**Table 3:** Comparison of PV detection in matching BMFS and two-phase samples as measured by WHO algorithm

SL1	Two-phase +	Two-phase−	SL2	Two-phase+	Two-phase−	SL3	Two-phase+	Two-phase−
BMFS+	1	7	BMFS+	23	9	BMFS +	11	11
BMFS−	8	26	BMFS−	4	6	BMFS−	3	17
OR (95% CI)	0.88 (0.32, 2.4)		OR (95% CI)	2.3 (0.69, 7.3)		OR (95% CI)	3.7 (1.0, 13)	
*p* value	0.80		*p* value	0.18		*p* value	0.035	

*PV* poliovirus, *BMFS* bag-mediated filtration system, *WHO (World Health Organization) algorithm* virus isolation on L20B and RD (human rhabdomyosarcoma) cells followed by ITD (intratypic differentiation), *SL1* Sabin-like PV type 1, *SL2* Sabin-like PV type 2, *SL3* Sabin-like PV type 3, *nd* not determined, *OR* odds ratio, *CI* 95% confidence intervals, *p value* calculated by the McNemar mid-p test

Dataset grouping did not result in a change in the statistical significance of SL1, SL2, or SL3 detection when comparing between BMFS and two-phase samples. SL1 and SL2 detection was not statistically different when comparing two-phase samples with the BMFS-1 and single BMFS samples dataset, the BMFS-2 and single BMFS samples dataset, or the BMFS concordant positive method dataset (*p* = 0.481, 1.0, and 0.80 for SL1, respectively, and *p* = 0.118, 0.057, and 0.18 for SL2, respectively). SL3 detection was statistically more frequent in the three BMFS datasets when compared individually to two-phase samples (*p* = 0.013, 0.035, and 0.035, respectively) (Tables 3, S7).

## Discussion

### Sample Collection and Processing

The goal of poliovirus environmental surveillance is to supplement AFP surveillance and assist in determining locations where PV is circulating or where it has been eliminated. It can also help with monitoring the elimination of SL PV after type-specific OPV cessation or complete cessation of OPV. PV (SL1, SL2, and/or SL3) was detected in a majority of BMFS and two-phase samples. The BMFS method demonstrated equivalent or greater PV detection when compared to WHO’s two-phase method based on the McNemar mid-p test (Table 3).

The feasibility of the BMFS method was demonstrated during implementation of this study for PV environmental surveillance. The method was successfully applied by staff from the national polio laboratory in Nairobi, Kenya (KEMRI) and the University of Pretoria laboratory personnel. Study sites were previously established by WHO, Kenya Ministry of Health, and KEMRI personnel for environmental surveillance. Due to security concerns at sites during the study, a bucket modification was developed to minimize field exposure. Transporting samples to a centralized processing location reduced the overall processing time when multiple sites were sampled on a single day by permitting parallel filtration. The bucket modification is recommended for security-compromised locations but, if security is not a concern, processing filters on-site may reduce the cost of transporting sewage or human-impacted residual waters if they are shipped.

MS2 was initially chosen as an internal control because it is non-pathogenic, found in the environment, and is similar to enteric viruses (Grabow 2001). In a previous study, with MS2 pre-seeded onto ViroCap filters, 10 L PV1-spiked Seattle influent wastewater filtered through these ViroCap filters, and preservatives added to the filters prior to elution, the average MS2 recovery (84.6%) was greater than the average PV1 recovery spiked into the 10 L influent wastewater (53.5%) (Fagnant et al. 2017). The erratic recovery of MS2 seen in this pilot study may have been due to various factors including deaggregation, MS2 presence in the environment, complications with the double agar layer assay, and/or seeding at different levels than anticipated. It is also possible the MS2 stock was left at room temperature for longer than prescribed by the protocol, thereby reducing the total PFUs seeded onto the filter. The relatively high frequency of PV detection in two-phase and BMFS field samples in comparison with the seeded MS2 may be due to a reduced robustness of laboratory-strain organisms when compared with environmental strains (Online Resource Table, S5) (Silverman and Nelson 2016). The inconsistent recovery of MS2 indicated its lack of suitability as an internal control and MS2 will not be included in future BMFS samples.

This study also showed that sample integrity could be maintained even when shipping filters to an out-of-country laboratory for processing and analysis, demonstrating the potential for use of this system in low-resource settings without local access to a processing laboratory. Samples were shipped on ice packs at 4 °C and a preservative mixture was added to the filters prior to shipment. For future BMFS studies, if filters are shipped for processing, the addition of preservatives is recommended, to stabilize virus and reduce bacterial and fungal growth in the case of cold-chain failure during shipment. The addition of preservatives has been found to increase virus survival in ViroCap filters held for up to 7 days at temperatures up to 25 °C (Fagnant et al. 2017). Future research to determine the effect of these preservatives on unconcentrated water samples should be examined for potential use with other sampling methods.

### Virus Detection

While there was an overall trend of increased SL detection by the BMFS method, there was statistically more frequent SL3 detection in BMFS than in two-phase samples, and no statistical difference in SL1 and SL2 detection (Fig. 2). The greater volumes processed with the BMFS (2.9 ± 0.1 L) compared to the two-phase method (0.5 L) may have contributed to the more frequent SL3 detection, the most frequent PV detected. The BMFS method includes a secondary concentration step utilizing PEG precipitation after filtration through ViroCap filters, which increases the concentration factor by tenfold. The two-phase method recommended by the WHO GPLN includes primary concentration only, which facilitates establishment of standardized environmental surveillance processing methods in a large number of laboratories. Additionally, incorporation of secondary concentration into the two-phase method would require additional sample manipulation (with increased chance of cross-contamination), longer processing time, and reduced sample volume to save in reserve. At the time of this study, the secondary concentration method would have required expensive equipment and therefore fewer laboratories would have the capability to implement it. Recent studies have improved the BMFS secondary concentration method to lower the centrifuge speed and eliminate overnight shaking at 4 °C (Falman et al. 2019), and field validation has been completed.

The differences observed between the BMFS and two-phase PV detection results could also be due to the variable recovery rates between the three PV types. Previous efforts have found PV recoveries of 38–48% for PV1, 56–89% for PV2, and 70–88% for PV3 during seeded studies with ViroCap filters and a variety of water sources (Fagnant et al. 2014). PV recovery could be affected by charge interactions as the viral capsid is negatively charged when at a pH below the isoelectric point. As SL3 has a lower isoelectric point (6.34) than SL1 (7.42) and SL2 (7.18), SL3 likely has a higher affinity to the positively charged ViroCap filters during filtration (Thomassen et al. 2013). SL1 detection in BMFS (8 of 42 samples) and two-phase samples (9 of 42 samples) and SL2 detection in BMFS (32 of 42 samples) and two-phase samples (27 of 42 samples) were not statistically different (*p* = 0.80 and 0.18, respectively). The stringent negative control process of sample amplification in cell culture, followed by PCR provides confidence in positive samples being true positives. While SL3 detection was more frequent using the BMFS than two-phase method, three samples were discordant in favor of two-phase.

The frequency of SL2 detection in both BMFS and two-phase samples can provide an important baseline to compare SL2 prevalence and persistence after the switch from trivalent OPV to bivalent OPV. The frequency of SL2 detection was comparable to other settings (Esteves-Jaramillo et al. 2014; Nakamura et al. 2015; Wahjuhono et al. 2014; Wang et al. 2014). This may be due to the high rate of PV2 shedding (88%), compared to PV1 (42%) and PV3 (58%) shedding 1 week after OPV use (Laassri et al. 2005) as demonstrated by frequent PV2 detection within 3 weeks after OPV use (*n* = 10) compared to prior to OPV use (*n* = 32) (SL1: 40% vs. 38%, SL2: 90% vs. 84%, and SL3: 60% vs. 59% in BMFS and two-phase samples, respectively).

Frequency of PV detection by BMFS varied among the three methods (Table 2). Similar PV detection rates between the WHO algorithm (WHO 2015) and ICC-RT-PCR suggest that both methods are able to detect PV even though ICC-RT-PCR is not specifically targeted towards PV. The greater frequency of PV detection by the WHO algorithm and ICC-RT-PCR compared to direct RT-PCR is likely due to the increased volume assayed and virus amplification by cell culture. After concentration, 3 mL was inoculated into cell culture in ICC-RT-PCR. In comparison, 1 mL of the concentrate was used for nucleic acid extraction, and 5% of the extracted volume was used as the input in direct RT-PCR.

Non-polio enterovirus (NPEV) was detected in 87% of samples negative for PV, and 22% of samples positive for PV (Fig. 2). The WHO virus isolation algorithm is designed for PV detection, and if no PV is isolated, NPEV can be determined via two routes. NPEV presence can be determined if cytopathic effects are present solely in the RD cell culture flask, as this indicates NPEV presence and no isolated PV. Therefore, when PV is detected in a flask, it masks NPEV in that flask. NPEV can also be reported from ITD results if the panEV assay is positive and all PV assays are negative. It is possible the more frequent detection of any PV in BMFS samples (37 of 42) compared to two-phase samples (32 of 42) may have contributed to the less frequent detection of NPEV in BMFS samples (13 of 42) compared to two-phase samples (24 of 42).

Study limitations existed including those related to sample collection and processing. While BMFS and two-phase samples were collected within 5 min and a 1-meter radius of each other, there is natural variation in viral distribution that could affect the results and lead to discrepancies between matched samples. Furthermore, the full sample was not assayed during detection. If a low virus concentration was present in the sample, then it is possible the virus was not present in the assayed portion of the sample. This may also partially explain discordance in virus detection. Further, sample collection occurred in the late morning or early afternoon. If samples were collected earlier in the day as recommended by the WHO GPLN (*e.g.,* by 9 a.m.), the detection rates may potentially have been greater. Due to the study design and laboratory capacity, BMFS samples were processed at the University of Pretoria and BMFS and two-phase samples were analyzed by the WHO algorithm at CDC. The shipping and storage times and conditions may have affected the detection of PV. Finally, as only SL PV was detected in this study, additional evaluation in WPV endemic regions is necessary to show BMFS capacity to detect circulating WPV (Zhou et al. 2018).

## Conclusions

This study demonstrated the feasibility of using BMFS for monitoring PV in environmental samples. Advantages of the BMFS include the ability to ship filters rather than unconcentrated wastewater samples and the ability to process large sample volumes. A disadvantage of the BMFS is a longer on-site processing time, leading to extended exposure at potentially unsafe sampling sites necessitating a bucket protocol, which allows for safer and more efficient sampling and processing. The increased frequency of SL3 detection in BMFS samples compared to the two-phase suggests that the BMFS method can result in greater SL3 detection likely due to the effective volume assayed (870 mL) and isoelectric point. BMFS requires secondary concentration to reach the target volume used for analysis by the WHO virus isolation algorithm (10–12 mL), whereas the two-phase method already results in this target volume. Therefore, there are limited benefits for use of secondary concentration with the two-phase method and the GPLN determined that there is no sufficient benefit to warrant global implementation. In an increasingly polio-free world, a cost–benefit analysis may be helpful to determine if any potential increase in sensitivity of PV detection would outweigh the burden of a secondary concentration step in the two-phase method, and if a new procedure is in line with future changes in the PV detection algorithm. Further validation would need to be conducted and run in parallel with the two-phase method to explore any alternative detection method.

## Electronic supplementary material

Below is the link to the electronic supplementary material.

Electronic supplementary material 1 (DOCX 64 kb)

## References

[CR1] Adams MH (1959). Bacteriophages.

[CR2] Anis E, Kopel E, Singer S, Kaliner E, Moerman L, Moran-Gilad J (2013). Insidious reintroduction of wild poliovirus into Israel, 2013. Eurosurveillance.

[CR3] Asghar H, Diop OM, Weldegebriel G, Malik F, Shetty S, El Bassioni L (2014). Environmental surveillance for polioviruses in the global polio eradication initiative. The Journal of Infectious Diseases.

[CR4] Borus P, Onuekwusi I, Maina C, Nyangao J, Dybdahl-Sissoko N, Penaranda S (2015). Environmental surveillance in Kenya. African Health Monitor.

[CR5] Chowdhary R, Dhole TN (2008). Interrupting wild poliovirus transmission using oral poliovirus vaccine: Environmental surveillance in high-risks area of India. Journal of Medical Virology.

[CR6] Cowger TL, Burns CC, Sharif S, Gary HE, Iber J, Henderson E (2017). The role of supplementary environmental surveillance to complement acute flaccid paralysis surveillance for wild poliovirus in Pakistan—2011–2013. PLoS ONE.

[CR7] Deshpande JM, Shetty SJ, Siddiqui ZA (2003). Environmental surveillance system to track wild poliovirus transmission. Applied and Environmental Microbiology.

[CR8] Diop OM, Asghar H, Gavrilin E, Moeletsi NG, Benito GR, Paladin F, Quddus A (2017). Virologic monitoring of poliovirus type 2 after oral poliovirus vaccine type 2 withdrawal in April 2016—worldwide, 2016–2017. MMWR: Morbidity and Mortality Weekly Report.

[CR9] El Bassioni L, Barakat I, Nasr E, de Gourville EM, Hovi T, Blomqvist S (2003). Prolonged detection of indigenous wild polioviruses in sewage from communities in Egypt. American Journal of Epidemiology.

[CR10] Esteves-Jaramillo A, Estivariz CF, Penaranda S, Richardson VL, Reyna J, Coronel DL (2014). Detection of vaccine-derived polioviruses in mexico using environmental surveillance. Journal of Infectious Diseases.

[CR11] Fagerland MW, Lydersen S, Laake P (2013). The McNemar test for binary matched-pairs data: mid-p and asymptotic are better than exact conditional. BMC Medical Research Methodology.

[CR12] Fagnant CS, Beck NK, Yang M-F, Barnes KS, Boyle DS, Meschke JS (2014). Development of a novel bag-mediated filtration system for environmental recovery of poliovirus. Journal of Water and Health.

[CR13] Fagnant CS, Kossik AL, Zhou NA, Sánchez-Gonzalez L, Falman JC, Keim EK (2017). Use of preservative agents and antibiotics for increased poliovirus survival on positively charged filters. Food and Environmental Virology.

[CR14] Fagnant CS, Sánchez-Gonzalez LM, Zhou NA, Falman JC, Eisenstein M, Guelig D (2018). Improvement of the bag-mediated filtration system for sampling wastewater and wastewater-impacted waters. Food and Environmental Virology.

[CR15] Falman JC, Fagnant-Sperati CS, Kossik AL, Boyle DS, Meschke JS (2019). Evaluation of secondary concentration methods for poliovirus detection in wastewater. Food and Environmental Virology.

[CR16] Gardner TJ, Diop OM, Jorba J, Chavan S, Ahmed J, Anand A (2018). Surveillance to track progress toward polio eradication—worldwide, 2016–2017. MMWR: Morbidity and Mortality Weekly Report.

[CR17] Gerloff N, Sun H, Mandelbaum M, Maher C, Nix WA, Zaidi S (2018). Diagnostic assay development for poliovirus eradication. Journal of Clinical Microbiology.

[CR18] Grabow WOK (2001). Bacteriophages: Update on application as models for viruses in water. Water SA.

[CR19] Grabow WOK, Botma KL, de Villiers JC, Clay CG, Erasmus B (1999). Assessment of cell culture and polymerase chain reaction procedures for the detection of polioviruses in wastewater. Bulletin of the World Health Organization.

[CR20] Hovi T, Blomqvist S, Nasr E, Burns CC, Sarjakoski T, Ahmed N (2005). Environmental surveillance of wild poliovirus circulation in Egypt–balancing between detection sensitivity and workload. Journal of Virological Methods.

[CR21] Hovi T, Shulman LM, van der Avoort H, Deshpande J, Roivainen M, Gourville DE, E. M. (2012). Role of environmental poliovirus surveillance in global polio eradication and beyond. Epidemiology and Infection.

[CR22] Kilpatrick DR, Yang C-F, Ching K, Vincent A, Iber J, Campagnoli R (2009). Rapid group-, serotype-, and vaccine strain-specific identification of poliovirus isolates by real-time reverse transcription-PCR using degenerate primers and probes containing deoxyinosine residues. Journal of Clinical Microbiology.

[CR23] Laassri M, Lottenbach K, Belshe R, Wolff M, Rennels M, Plotkin S, Chumakov K (2005). Effect of different vaccination schedules on excretion of oral poliovirus vaccine strains. Journal of Infectious Diseases.

[CR24] Lopalco PL (2017). Wild and vaccine-derived poliovirus circulation, and implications for polio eradication. Epidemiology and Infection.

[CR25] Maes EF, Diop OM, Jorba J, Chavan S, Tangermann RH, Wassilak SGF (2017). Surveillance systems to track progress toward polio eradication—worldwide, 2015–2016. MMWR Morbidity and Mortality Weekly Report.

[CR26] Malherbe H, Strickland-Cholmley M (1980). Viral cytopathology.

[CR28] Manor Y, Blomqvist S, Sofer D, Alfandari J, Halmut T, Abramovitz B (2007). Advanced environmental surveillance and molecular analyses indicate separate importations rather than endemic circulation of wild type 1 poliovirus in Gaza district in 2002. Applied and Environmental Microbiology.

[CR27] Manor Y, Handsher R, Halmut T, Neuman M, Bobrov A, Rudich H (1999). Detection of poliovirus circulation by environmental surveillance in the absence of clinical cases in israel and the palestinian authority. Journal of Clinical Microbiology.

[CR29] McNemar Q (1947). Note on the sampling error of the difference between correlated proportions or percentages. Psychometrika.

[CR30] Mendez II, Hermann LL, Hazelton PR, Coombs KM (2000). A comparative analysis of Freon substitutes in the purification of reovirus and calicivirus. Journal of Virological Methods.

[CR31] Nakamura T, Hamasaki M, Yoshitomi H, Ishibashi T, Yoshiyama C, Maeda E (2015). Environmental surveillance of poliovirus in sewage water around the introduction period for inactivated polio vaccine in Japan. Applied and Environmental Microbiology.

[CR32] Nijst OEM, Mouthaan JJ, Mekkes DR, Jusic E, van der Avoort HGAM, Metz B (2013). Rapid and accurate identification of poliovirus strains used for vaccine production. Journal of Virological Methods.

[CR33] Shukla D, Kumar A, Srivastava S, Idris MZ, Dhole TN (2013). Environmental surveillance of enterovirus in Northern India using an integrated shell vial culture with a semi-nested RT PCR and partial sequencing of the VP1 gene. Journal of Medical Virology.

[CR34] Silverman AI, Nelson KL (2016). Modeling the endogenous sunlight inactivation rates of laboratory strain and wastewater *E. coli* and enterococci using biological weighting functions. Environmental Science & Technology.

[CR35] Snider CJ, Diop OM, Burns CC, Tangermann RH, Wassilak SGF (2016). Surveillance systems to track progress toward polio eradication—worldwide, 2014–2015. MMWR: Morbidity and Mortality Weekly Report.

[CR36] Thomassen YE, van Eikenhorst G, van der Pol LA, Bakker WAM (2013). Isoelectric point determination of live polioviruses by capillary isoelectric focusing with whole column imaging detection. Analytical Chemistry.

[CR37] US EPA. (2000). Method 1602: Male-specific (F+) and somatic coliphage in water by single agar layer (SAL) procedure EPA 821-R-01–029. EPA Office of Water.

[CR38] Wahjuhono WFNG, Revolusiana W, Sundoro J, Mardani T, Ratih WU, et al. (2014). 2014 Switch from oral to inactivated poliovirus vaccine in Yogyakarta Province, Indonesia: Summary of coverage, immunity, and environmental surveillance. Journal of Infectious Diseases.

[CR39] Wang H, Tao Z, Li Y, Lin X, Yoshida H, Song L (2014). Environmental surveillance of human enteroviruses in Shandong Province, China, 2008 to 2012: Serotypes, temporal fluctuation, and molecular epidemiology. Applied and Environmental Microbiology.

[CR40] WHO. (2003). Guidelines for environmental surveillance of poliovirus circulation. Department of Vaccines and Biologicals. World Health Organization, Geneva, Switzerland. Retrieved April 10, 2018, from https://apps.who.int/iris/bitstream/handle/10665/67854/WHO_V-B_03.03_eng.pdf;jsessionid=F066B374A6E1002F5BB0F27D57A212D1?sequence=1.

[CR41] WHO. (2013). Polio eradication & endgame strategic plan 2013–2018. World Health Organization. Retrieved April 10, 2018, from https://polioeradication.org/wp-content/uploads/2016/07/PEESP_EN_A4.pdf.

[CR42] WHO. (2015). Guidelines on environmental surveillance for detection of polioviruses. Retrieved April 10, 2018, from https://polioeradication.org/wp-content/uploads/2016/07/GPLN_GuidelinesES_April2015.pdf.

[CR43] Yadav PR, Tyagi R (2008). Cell culture.

[CR44] Zhou NA, Fagnant-Sperati CS, Shirai JH, Sharif S, Zaidi SZ, Rehman L (2018). Evaluation of the bag-mediated filtration system as a novel tool for poliovirus environmental surveillance: Results from a comparative field study in Pakistan. PLoS ONE.

