# Supplementary material for: Feasibility of the Bag-Mediated Filtration System for Environmental Surveillance of Poliovirus in Kenya
Source: Food Environ Virol. 2019 Nov 2;12(1):35–47. doi: 10.1007/s12560-019-09412-1 (PMC7052051; doi:10.1007/s12560-019-09412-1)
Supplement: Supplementary file 1 — Electronic supplementary material 1 (DOCX 64 kb) [file 12560_2019_9412_MOESM1_ESM.docx]

ELECTRONIC SUPPLEMENTARY MATERIAL

*Feasibility and applicability of the bag-mediated filtration system for enhanced environmental surveillance of poliovirus in Kenya using three analysis methods*

Nicolette A. Zhou^1†^, Christine S. Fagnant-Sperati^1†^, Evans Komen^2^, Benlick Mwangi^2^, Johnstone Mukubi^2^, James Nyangao^2^, Joanne Hassan^2^, Agnes Chepkurui^2^, Caroline Maina^3^, Walda B. van Zyl^4^, Peter N. Matsapola^4^, Marianne Wolfaardt^4^, Fhatuwani B. Ngwana^4^, Stacey Jeffries-Miles^5^, Angela Coulliette-Salmond^6^, Silvia Peñaranda^6^, Jeffry H. Shirai^1^, Alexandra L. Kossik^1^, Nicola K. Beck^1^, Robyn Wilmouth^7^, David S. Boyle^7^, Cara C. Burns^6^, Maureen B. Taylor^4^, Peter Borus^2^, J. Scott Meschke^1^*

^1^ Department of Environmental and Occupational Health Sciences, University of Washington; 4225 Roosevelt Way NE, Suite 100, Seattle, WA 98105, USA

^2^ Centre for Viral Research, Kenya Medical Research Institute; Mbagathi Road, P.O. Box 54628, Nairobi 00200, Kenya

^3^ Kenya Ministry of Health; Afya House, Cathedral Road, P.O. Box 30016, Nairobi 00100, Kenya

^4^ Department of Medical Virology, University of Pretoria; Room 3-33 Pathology Building, Cnr Beatrix Str & Dr Savage Road, Pretoria 0002, South Africa

^5^ IHRC, Inc.; 2 Ravinia Drive, Suite 1200, Atlanta, GA 30346, USA

^6^ Division of Viral Diseases, Centers for Disease Control and Prevention; 1600 Clifton Road NE, Mailstop H17-6, Atlanta, GA 30329, USA

^7^ PATH; 2201 Westlake Ave, Suite 200, Seattle, WA 98121, USA

^†^ equal contributions towards authorship

*corresponding author: email: jmeschke@uw.edu; phone: +1-206-221-5470

*Submitted to Food and Environmental Virology*

**Table of contents**

**MS2 recovery experiments** 2

**Fig. S1** Infectious MS2 recovery from ViroCap filters after storage at 4°C. 2

**Table S1.** Primers and probes used for detection of SL1, SL2, SL3, WPV1, and WPV3 2

**Table S2.** Real-time RT-PCR programs used for poliovirus detection 2

**Table S3.** ICC-RT-PCR cell lines and media used 2

**Table S4.** BMFS sampling scheme 2

**Table S5.** Comparison of PV and MS2 detection in matched two-phase and BMFS samples. 2

**Table S6.** Comparison of PV detection in BMFS samples as measured by three different detection methods 2

**Table S7.** Comparison of PV detection in BMFS samples analyzed by ICC-RT-PCR 2

**Table S8.** Comparison of PV detection in matching BMFS and two-phase samples as measured by WHO algorithm 2

**References** 2

# **MS2 recovery experiments**

*Methods*

Approximately 10^5^ PFU of MS2 (ATCC 15597-B1) were seeded to 175 mL 1x phosphate-buffered saline (PBS), agitated using a vortex (30 seconds), and passed through the ViroCap filter inlet using a peristaltic pump. The filtrate was collected and discarded. Filters were stored for 0, 1, 2, 4, or 7 days at 4°C, and two filters were tested for each time point.

Filters were eluted as previously described, using 175 mL sterile 1.5% beef extract, 0.05 M glycine, pH 9.5 (Fagnant et al. 2014). The eluent was added to the filter inlet, let to stand 30 minutes, and recovered via the outlet. The recovered eluate was then pH-adjusted to 7.0-7.5 using 5 M HCl and 5 M NaOH. Infectious MS2 was enumerated by the double agar layer method on *E. coli* F-amp host (ATCC 70081) (Adams 1959). Briefly, 100 µL sample dilutions in PBS and 100 µL log-phase *E. coli* F-amp in nutrient broth were added to 6-8 mL molten bactoagar (0.7% bactoagar, 0.5% sodium chloride) and mixed. The bactoagar was then poured onto a 100 mm tryptic soy agar plate and let to solidify. Plates were incubated at 37°C for 18-20 hours and plaques were counted. Relevant dilutions were plated in duplicate, and recovery was calculated by dividing the recovered count by the known seeded count.

*Results and Discussion*

MS2 recovery averaged 74.9%, 63.7%, 54.3%, 55.3%, and 69.9% after 0, 1, 2, 4, and 7 days, respectively (Fig. S1). The relatively high MS2 recovery after one day indicates filters could be stored for up to 24 hours after MS2 seeding prior to filtration, while still anticipating greater than 50% recovery of infectious MS2.

# **Fig. S1** Infectious MS2 recovery from ViroCap filters after storage at 4°C. *n*=2 per filter storage time.

# **Table S1.** Primers and probes used for detection of SL1, SL2, SL3, WPV1, and WPV3

| Primer | Sequence (5🡪3) | Orientation | Position |
| --- | --- | --- | --- |
| *Centers for Disease Control and Prevention* ^a^ | | | |
| **Quadruplex** |  |  |  |
| Pan-EV PCR-1 | GCGATTGTCACCATWAGCAGYCA | Reverse | 603–581 |
| Pan-EV PCR-2 | GGCCCCTGAATGCGGCTAATCC | Forward | 458–480 |
| Pan-EV Probe | VIC-CCGACTACTTTGGGWGTCCGTGT-TAMRA |  | 546–568 |
| Sabin 1 PCR-1 | CCACTGGCTTCAGTGTTT | Reverse | 2600–2583 |
| Sabin 1 PCR-2 | AGGTCAGATGCTTGAAAGC | Forward | 2505–2523 |
| Sabin 1 Probe A4 | CY5-CGCCCCCACCGTTTCACGGA-BHQ3 |  | 2559–2540 |
| Sabin 2 PCR-1 | CGGCTTTGTGTCAGGCA | Reverse | 2595–2579 |
| Sabin 2 PCR-2 | CCGTTGAAGGGATTACTAAA | Forward | 2525–2544 |
| Sabin 2 Probe | FAM-ATTGGTTCCCCCGACTTCCACCAAT-BHQ1 |  | 2550–2572 |
| Sabin 3 PCR-1 | TTAGTATCAGGTAAGCTATC | Reverse | 2591–2572 |
| Sabin 3 PCR-2 | AGGGCGCCCTAACTTT | Forward | 2537–2552 |
| Sabin 3 Probe | ROX-TCACTCCCGAAGCAACAG-BHQ2 |  | 2554–2571 |
| **Pan Poliovirus** |  |  |  |
| Pan-PV PCR-1 | GGAGCTCCGGGTGGGAYRTACATIATYTGRTAIAC | Reverse | 2978–2956 |
| Pan-PV PCR-2 | TTGGAGTTCTTCACITAITCIMGITTYGAYATG | Forward | 2876–2895 |
| Pan-PV Probe | FAM-TGRTTNARIGCRTGICCRTTRTT-BHQ1 |  | 2957–2935 |
| *University of Pretoria* ^b^ | | | |
| SABIN1-F | TCCCTTTGACTTAAGTACAAA | Forward | 1904-1924 |
| POLIO1-R | GATCCTGCCCAGTGTGTGTAG | Reverse | 2083-2063 |
| POLIO1-TM | FAM-AGGGTTCGGTTAAGTGACAAACCACATAC-BBQ |  | 1950-1978 |
| SABIN2-F | AAGGAATTGGTGACATGATTGAGG | Forward | 2480-2503 |
| SABIN2-R | CTCGGCTTTGTGTCAGGC | Reverse | 2579-2562 |
| SABIN2-TM | FAM-TGGAAGTCGGGGGAACCAATGC-BBQ |  | 2551-2530 |
| SABIN3-F | AATGACCAGATTGGTGATTCCTTG | Forward | 3134-3157 |
| SABIN3-R | GTAAATGCGGACTTTGGAGGTTACT | Reverse | 3253-3229 |
| SABIN3-TM | FAM-TGTGATCATTGACAACACGAACTGCCAA-BBQ |  | 3218-3191 |

SL1, Sabin-like poliovirus type 1; SL2, Sabin-like poliovirus type 2; SL3, Sabin-like poliovirus type 3; ITD, intratypic differentiation

^a^ The Centers for Disease Control and Prevention used the Poliovirus ITD 4.0/4.1 rRT-PCR Kit

^b^ University of Pretoria used the Poliovirus ITD 4.0/4.1 rRT-PCR Kit for detection of WPV1 and WPV3 as described for the Poliovirus ITD 5.0 rRT-PCR Kit by Gerloff et al. 2018, and primers and probes from Nijst et al. 2013 for detection of SL1, SL2, and SL3

# **Table S2.** Real-time RT-PCR programs used for poliovirus detection

|  | Centers for Disease Control and Prevention ^a^ | |  | University of Pretoria ^b^ | | | | | | | |  |
| --- | --- | --- | --- | --- | --- | --- | --- | --- | --- | --- | --- | --- |
|  | All primer sets | |  | SL1 | |  | SL2 | |  | SL3 | |  |
|  | T  (°C) | time |  | T (°C) | time |  | T (°C) | time |  | T (°C) | time |  |
| reverse transcription | 42 | 45 min |  | 50 | 45 min |  | 50 | 45 min |  | 50 | 45 min |  |
| PCR activation | 95 | 3 min |  | 95 | 15 min |  | 95 | 15 min |  | 95 | 15 min |  |
| cycles | 40 cycles | |  | 45 cycles | | | | | | | | |
| denaturation | 95 | 24 sec |  | 95 | 5 sec |  | 95 | 5 sec |  | 95 | 5 sec |  |
| annealing | 47 | 30 sec |  | 55 | 10 sec |  | 58 | 15 sec |  | 56 | 15 sec |  |
| extension | 65 | 24 sec |  | 72 | 10 sec |  | 72 | 5 sec |  | 72 | 5 sec |  |
| cool down | - | - |  | 40 | 30 sec |  | 40 | 30 sec |  | 40 | 30 sec |  |

SL1, Sabin-like poliovirus type 1; SL2, Sabin-like poliovirus type 2; SL3, Sabin-like poliovirus type 3; WPV1, wild poliovirus type 1; WPV3, wild poliovirus type 3; T, temperature; ITD, intratypic differentiation

^a^ Poliovirus ITD 4.0/4.1 rRT-PCR Kit

^b^ University of Pretoria used the Poliovirus ITD 4.0/4.1 rRT-PCR Kit for detection of WPV1 and WPV3

# **Table S3.** ICC-RT-PCR cell lines and media used

| Cell line | Obtained from | Citation | Propagation media | Wash  media | Maintenance  media |
| --- | --- | --- | --- | --- | --- |
| PLC/  PRF/5 | ECACC 85061113 | Alexander et al. 1976 | E-MEM Pen/Strep 8% FCS Hepes Buffer Tylosin | E-MEM Pen/Strep | E-MEM Pen/Strep Amphotericin B 2% FCS |
| L20B | CDC via the NICD | Pipkin et al. 1993; Wood and Hull 1999 | E-MEM Pen/Strep 5% FCS Hepes Buffer Tylosin | E-MEM Pen/Strep | E-MEM Pen/Strep Amphotericin B 5% FCS |
| BGM | ECACC 90092601 | Dahling et al. 1974 | E-MEM Pen/Strep 5% FCS | E-MEM Pen/Strep | E-MEM Pen/Strep Amphotericin B 0.5% FCS |

ICC-RT-PCR, integrated cell culture-real-time RT-PCR (reverse-transcription polymerase chain reaction) with PLC/PRF/5, L20B, and BGM (buffalo green monkey) cell lines; ECACC, European Collection of Cell Cultures (Salisbury, UK); CDC, Centers for Disease Control and Prevention; NICD, National Institute for Communicable Diseases (Sandringham, SA); E-MEM, Eagle's minimal essential medium with Earle's salts and L-glutamine (Gibco, Carlsbad, CA, USA); Pen/Strep, 100 U/mL penicillin and 100 µg/mL streptomycin (BioWhittaker® Pen/Strep, Lonza, Verviers, Belgium); FCS, fetal calf serum (FBS Superior, Biochrom, Berlin, Germany); Hepes Buffer, 10 mM Hepes buffer solution (Gibco); Tylosin, 0.008 mg/mL Tylosin solution (Sigma-Aldrich Co., St. Louis, MO, USA); Pen/Strep Amphotericin B, 100 U/mL penicillin, 100 µg/mL streptomycin, and 0.0025 µg/mL amphotericin B (BioWhittaker Pen/Strep Amphotericin B [100x], Lonza)

# **Table S4.** BMFS sampling scheme

| Sample collection day (dd/mm/yyyy) | Samples collected | Sequential samples collected | Number of sites sampled | Bucket modification conducted | Collection time (hours) ^d^ | Time to preservative addition (days) ^f^ | Time to shipping (days) ^h^ | Shipping time (days) | Time to elution (days) ^k^ | Time to secondary concentration (days) ^m^ | Overall processing time (days) ^p^ |
| --- | --- | --- | --- | --- | --- | --- | --- | --- | --- | --- | --- |
| 14/04/2015 | 4 | yes | 2 ^a^ | no | 3.5 | 21 | 22 ^i^ |  | 0 | 4 | 29 ^q^ |
| 28/04/2015 | 6 | yes | 3 ^b^ | no | 6.2 | 7 | 8 ^i^ | 2 ^i^ | 0/3 ^l^ | 4/1 ^n^ | 15 |
| 04/05/2015 | 2 | yes | 1 ^c^ | no | 1.3 | 1 | 2 ^i^ |  | 3 | 1 | 9 |
| 13/05/2015 | 8 | yes | 4 | yes | 6.3 | 5 | 6 | 2 | 0 | 1 | 10 |
| 26/05/2015 | 8 | yes | 4 | yes | 5.3 | 2/3 ^g^ | 7 | 7 ^j^ | 1 | 4 | 15 |
| 16/06/2015 | 4 | no | 4 | yes | 4.8 | 2 | 3 | 2 | 0 | 4 | 11 |
| 30/06/2015 | 4 | no | 4 | yes | 3.6 | 1 | 8 | 2 | 3 | 1 | 15 |
| 21/07/2015 | 4 | no | 4 | yes | 4.5 | 2 | 6 | 2 | 1 | 1 | 11 |
| 28/07/2015 | 4 | no | 4 | yes | 4.3 | 1 | 6 | 2 | 2 | 5 | 16 |
| 11/08/2015 | 4 | no | 4 | yes | 4.4 | 2 | 7 | 2 | 1 | 4 | 15 |
| 25/08/2015 | 4 | no | 4 | yes | 5.0 | 3 | 8 | 2 | 4 | 1 | 16 |
| 07/09/2015 | 4 | no | 4 | yes | 4.2 | 1 | 2 | 2 | 0 | 4 | 10 |
| 12 sampling days | 56 BMFS samples collected | Collected during first 5 sampling days | 9 sampling days where all 4 sites sampled | Bucket modification used during last 9 sampling days | Average collection time was 4.7 hours when 4 sites sampled on the same day ^e^ | Occurred within 3 days for 68% of filters | Average hold time was 6 days for 79% of filters | 2 days for 90% of shipping events | Occurred within 4 days of filter receipt | Occurred within 5 days of elution ^o^ | Median time was 15±1.3 days |

BMFS, bag-mediated filtration system; ^a^ samples only collected from Kibera and Starehe, as the method was new to the field technicians and a scaled sampling was desired; ^b^ planned sampling event at Kibera was postponed to one week later due to the time of day and traffic considerations; ^c^ make-up sampling day for the postponed sample from Kibera; ^d^ time from the beginning of first sample collection to arrival back at KEMRI; ^e^ collection time does not include filtration time for these 9 sampling days; ^f^ between sample collection and preservative addition; ^g^ preservatives added to Kibera samples within 2 days, while preservatives were added to Starehe, Eastleigh A, and Eastleigh B samples within 3 days; ^h^ between sample collection and shipment to the University of Pretoria; ^i^ samples from the first three sampling days shipped together. The delay in shipping was due to incorporation of new routines, procurement of shipment boxes, and coordination with the courier;  ^j^ shipping time was seven days due to a courier mistake in which the filters were detoured rather than flown directly from Kenya to South Africa; ^k^ between sample receipt at the University of Pretoria and filter elution; ^l^ filter elution was completed for Starehe samples on the day of receipt, while elution of Kibera, Eastleigh A, and Eastleigh B samples was completed three days after filter receipt; ^m^ between filter elution and initiation of secondary concentration; ^n^ secondary concentration was completed on the same day for samples from all four sites, though filter elution was completed for Starehe samples three days prior to filter elution for the samples from the other sites; ^o^ delays in completion of secondary concentration was due to the overnight step required. Therefore, when elution occurred on a Thursday, secondary concentration was delayed until Monday; ^p^ between sample collection and completion of secondary concentration; ^q^ the time delay was due to logistical challenges with shipping (7% of filters)

# **Table S5.** Comparison of PV and MS2 detection in matched two-phase and BMFS samples.

| Sample collection day (dd/mm/yyyy) | Site | Two-phase | | |  | BMFS | | | |
| --- | --- | --- | --- | --- | --- | --- | --- | --- | --- |
|  |  | SL1^a^ | SL2^a^ | SL3^a^ |  | SL1^a^ | SL2^a^ | SL3^a^ | MS2^b^ |
| 14/04/2015 | Kibera | 0 | 0 | 0 |  | 1 | 1 | 1 | 478 |
| 14/04/2015 | Kibera |  |  |  |  | 0 | 1 | 1 | 33 |
| 14/04/2015 | Starehe | 0 | 0 | 1 |  | 0 | 1 | 1 | 128 |
| 14/04/2015 | Starehe |  |  |  |  | 0 | 1 | 1 | 316 |
| 28/4/2015 | Starehe | 1 | 0 | 1 |  | 0 | 0 | 1 | 3.8 |
| 28/4/2015 | Starehe |  |  |  |  | 0 | 1 | 1 | 247 |
| 28/4/2015 | Eastleigh A | 0 | 0 | 0 |  | 1 | 1 | 0 | 77.3 |
| 28/4/2015 | Eastleigh A |  |  |  |  | 1 | 0 | 0 | 74.3 |
| 28/4/2015 | Eastleigh B | 0 | 0 | 0 |  | 0 | 0 | 1 | 0.7 |
| 28/4/2015 | Eastleigh B |  |  |  |  | 0 | 0 | 1 | 0.7 |
| 05/04/2015 | Kibera | 0 | 1 | 1 |  | 1 | 1 | 1 | 430 |
| 05/04/2015 | Kibera |  |  |  |  | 1 | 1 | 1 | 244 |
| 13/05/2015 | Kibera | 0 | 0 | 0 |  | 0 | 0 | 1 | 0.7 |
| 13/05/2015 | Kibera |  |  |  |  | 0 | 0 | 1 | 1.4 |
| 13/05/2015 | Starehe | 1 | 1 | 0 |  | 1 | 0 | 1 | 9.5 |
| 13/05/2015 | Starehe |  |  |  |  | 0 | 1 | 0 | 23.4 |
| 13/05/2015 | Eastleigh A | 0 | 1 | 0 |  | 1 | 1 | 1 | 134 |
| 13/05/2015 | Eastleigh A |  |  |  |  | 0 | 1 | 0 | 74.3 |
| 13/05/2015 | Eastleigh B | 1 | 0 | 1 |  | 0 | 1 | 0 | 113 |
| 13/05/2015 | Eastleigh B |  |  |  |  | 0 | 1 | 0 | 29.1 |
| 26/05/2015 | Kibera | 1 | 1 | 1 |  | 0 | 1 | 1 | 0 |
| 26/05/2015 | Kibera |  |  |  |  | 1 | 1 | 1 | 0.7 |
| 26/05/2015 | Starehe | 1 | 1 | 0 |  | 0 | 1 | 1 | 16.2 |
| 26/05/2015 | Starehe |  |  |  |  | 0 | 1 | 1 | 4 |
| 26/05/2015 | Eastleigh A | 0 | 0 | 0 |  | 0 | 1 | 0 | 73.6 |
| 26/05/2015 | Eastleigh A |  |  |  |  | 0 | 1 | 0 | 47.9 |
| 26/05/2015 | Eastleigh B | 0 | 0 | 0 |  | 1 | 1 | 1 | 8.8 |
| 26/05/2015 | Eastleigh B |  |  |  |  | 0 | 1 | 1 | 2.02 |
| 16/06/2015 | Kibera | 0 | 1 | 0 |  | 1 | 0 | 1 | 0.7 |
| 16/06/2015 | Starehe | 0 | 1 | 0 |  | 1 | 1 | 0 | 0 |
| 16/06/2015 | Eastleigh A | 0 | 0 | 0 |  | 0 | 1 | 1 | 0.7 |
| 16/06/2015 | Eastleigh B | 0 | 1 | 1 |  | 0 | 0 | 0 | 1.4 |
| 30/06/2015 | Kibera | 0 | 1 | 0 |  | 1 | 1 | 1 | 6.8 |
| 30/06/2015 | Starehe | 0 | 0 | 1 |  | 0 | 1 | 1 | 0.7 |
| 30/06/2015 | Eastleigh A | 0 | 1 | 0 |  | 0 | 1 | 0 | 0.7 |
| 30/06/2015 | Eastleigh B | 1 | 1 | 0 |  | 1 | 1 | 0 | 0.7 |
| 21/07/2015 | Kibera | 0 | 1 | 1 |  | 0 | 1 | 1 | 25 |
| 21/07/2015 | Starehe | 0 | 1 | 0 |  | 0 | 1 | 1 | 499 |
| 21/07/2015 | Eastleigh A | 0 | 0 | 0 |  | 0 | 1 | 0 | 4.05 |
| 21/07/2015 | Eastleigh B | 0 | 1 | 1 |  | 0 | 1 | 1 | 0.7 |
| 28/07/2015 | Kibera | 0 | 1 | 0 |  | 0 | 1 | 0 | 0.1 |
| 28/07/2015 | Starehe | 0 | 1 | 1 |  | 0 | 1 | 1 | 23 |
| 28/07/2015 | Eastleigh A | 0 | 1 | 0 |  | 0 | 0 | 0 | 16 |
| 28/07/2015 | Eastleigh B | 0 | 0 | 0 |  | 0 | 0 | 0 | 120 |
| 08/11/2015 | Kibera | 0 | 1 | 0 |  | 0 | 1 | 0 | 31 |
| 08/11/2015 | Starehe | 0 | 1 | 1 |  | 1 | 1 | 1 | 11 |
| 08/11/2015 | Eastleigh A | 1 | 1 | 0 |  | 0 | 1 | 0 | 2.7 |
| 08/11/2015 | Eastleigh B | 0 | 1 | 0 |  | 0 | 1 | 1 | 8.8 |
| 25/08/2015 | Kibera | 0 | 1 | 0 |  | 1 | 1 | 0 | 6 |
| 25/08/2015 | Starehe | 1 | 1 | 1 |  | 0 | 1 | 1 | 0 |
| 25/08/2015 | Eastleigh A | 0 | 1 | 0 |  | 0 | 1 | 0 | 1 |
| 25/08/2015 | Eastleigh B | 0 | 1 | 1 |  | 0 | 1 | 1 | 0 |
| 09/07/2015 | Kibera | 0 | 1 | 0 |  | 0 | 1 | 0 | 1.4 |
| 09/07/2015 | Starehe | 0 | 1 | 1 |  | 0 | 1 | 0 | 50 |
| 09/07/2015 | Eastleigh A | 0 | 0 | 0 |  | 0 | 0 | 0 | 0 |
| 09/07/2015 | Eastleigh B | 1 | 0 | 0 |  | 0 | 1 | 1 | 10 |

PV, poliovirus; BMFS, bag-mediated filtration system; SL1, Sabin-like poliovirus type 1; SL2, Sabin-like poliovirus type 2; SL3, Sabin-like poliovirus type 3; ^a^ presence (1) or absence (0); samples were measured by WHO (World Health Organization) algorithm, using virus isolation on L20B and RD (human rhabdomyosarcoma) cells followed by ITD (intratypic differentiation); ^b^ percent recovery; samples were measured by double agar layer, and compared with 100% recovery of 10^5^ PFU seeded MS2

# **Table S6.** Comparison of PV detection in BMFS samples as measured by three different detection methods

| Comparison of PV detection in WHO algorithm and direct RT-PCR | | | | | | | | | | |
| --- | --- | --- | --- | --- | --- | --- | --- | --- | --- | --- |
| SL1 | WHO + | WHO - |  | SL2 | WHO + | WHO - |  | SL3 | WHO + | WHO - |
| direct + | 0 | 0 |  | direct + | 6 | 0 |  | direct + | 12 | 1 |
| direct - | 8 | 34 |  | direct - | 26 | 10 |  | direct - | 10 | 19 |
| OR (CI) | nd | |  | OR (CI) | nd | |  | OR (CI) | 10 (1.3, 78) | |
| *p*-value | 0.0039 |  |  | *p*-value | 1.49x10^-8^ | |  | *p*-value | 0.0063 |  |
| Comparison of PV detection in WHO algorithm and ICC-RT-PCR | | | | | | | | | | |
| SL1 | WHO + | WHO - |  | SL2 | WHO + | WHO - |  | SL3 | WHO + | WHO - |
| ICC + | 5 | 9 |  | ICC + | 31 | 5 |  | ICC + | 19 | 9 |
| ICC - | 3 | 25 |  | ICC - | 1 | 5 |  | ICC - | 3 | 11 |
| OR (CI) | 0.33 (0.090, 1.2) | |  | OR (CI) | 0.2 (0.023, 1.7) | |  | OR (CI) | 0.33 (0.090, 1.2) | |
| *p*-value | 0.092 |  |  | *p*-value | 0.125 | |  | *p*-value | 0.092 |  |
| Comparison of PV detection in direct RT-PCR and ICC-RT-PCR | | | | | | | | | | |
| SL1 | ICC + | ICC - |  | SL2 | ICC + | ICC - |  | SL3 | ICC + | ICC - |
| direct + | 0 | 0 |  | direct + | 6 | 0 |  | direct + | 13 | 0 |
| direct - | 13 | 29 |  | direct - | 28 | 8 |  | direct - | 15 | 14 |
| OR (CI) | nd | |  | OR (CI) | nd | |  | OR (CI) | nd | |
| *p*-value | 0.00012 |  |  | *p*-value | 3.73x10^-9^ | |  | *p*-value | 3.05x10^-5^ | |

PV, poliovirus; BMFS, bag-mediated filtration system; WHO, World Health Organization; WHO algorithm measured by virus isolation on L20B and RD (human rhabdomyosarcoma) cells followed by ITD (intratypic differentiation); direct, direct real-time RT-PCR (reverse-transcription polymerase chain reaction); ICC, integrated cell culture-real-time RT-PCR with PLC/PRF/5, L20B, and BGM (buffalo green monkey) cell lines; SL1, Sabin-like PV type 1; SL2, Sabin-like PV type 2; SL3, Sabin-like PV type 3; nd, not determined; OR, odds ratio; CI, 95% confidence intervals; *p*-value, calculated by the McNemar mid-p test

# **Table S7.** Comparison of PV detection in BMFS samples analyzed by ICC-RT-PCR

| Comparison of PV detection after amplification on PLC/PRF/5 and L20B cell lines | | | | | | | | | | |
| --- | --- | --- | --- | --- | --- | --- | --- | --- | --- | --- |
| SL1 | PLC + | PLC - |  | SL2 | PLC + | PLC - |  | SL3 | PLC + | PLC - |
| L20B + | 4 | 6 |  | L20B + | 22 | 7 |  | L20B + | 14 | 5 |
| L20B - | 3 | 29 |  | L20B - | 4 | 9 |  | L20B - | 6 | 17 |
| OR (CI) | 0.5 (0.13, 2.0) | |  | OR (CI) | 0.57 (0.17, 2.0) | |  | OR (CI) | 1.2 (0.37, 3.9) | |
| *p*-value | 0.344 |  |  | *p*-value | 0.388 | |  | *p*-value | 0.774 |  |
| Comparison of PV detection after amplification on PLC/PRF/5 and BGM cell lines | | | | | | | | | | |
| SL1 | PLC + | PLC - |  | SL2 | PLC + | PLC - |  | SL3 | PLC + | PLC - |
| BGM + | 1 | 0 |  | BGM + | 12 | 1 |  | BGM + | 4 | 3 |
| BGM - | 6 | 35 |  | BGM - | 14 | 15 |  | BGM - | 16 | 19 |
| OR (CI) | nd | |  | OR (CI) | 14 (1.8, 106) | |  | OR (CI) | 5.3 (1.6, 18) | |
| *p*-value | 0.016 |  |  | *p*-value | 5.2x10^-4^ | |  | *p*-value | 0.0026 |  |
| Comparison of PV detection after amplification on L20B and BGM cell lines | | | | | | | | | | |
| SL1 | L20B + | L20B - |  | SL2 | L20B + | L20B - |  | SL3 | L20B + | L20B - |
| BGM + | 1 | 0 |  | BGM + | 12 | 1 |  | BGM + | 5 | 2 |
| BGM - | 9 | 32 |  | BGM - | 17 | 12 |  | BGM - | 14 | 21 |
| OR (CI) | nd | |  | OR (CI) | 17 (2.3, 128) | |  | OR (CI) | 7 (1.6, 31) | |
| *p*-value | 0.0020 |  |  | *p*-value | 7.63x10^-5^ | |  | *p*-value | 0.0024 | |

PV, poliovirus; BMFS, bag-mediated filtration system; ICC-RT-PCR, integrated cell culture-real-time RT-PCR (reverse-transcription polymerase chain reaction) with PLC/PRF/5, L20B, and BGM (buffalo green monkey) cell lines; SL1, Sabin-like PV type 1; SL2, Sabin-like PV type 2; SL3, Sabin-like PV type 3; nd, not determined; OR, odds ratio; CI, 95% confidence intervals; *p*-value, calculated by the McNemar mid-p test

# **Table S8.** Comparison of PV detection in matching BMFS and two-phase samples as measured by WHO algorithm

| Comparison of PV detection in BMFS-1 and single BMFS samples and two-phase samples | | | | | | | | | | |
| --- | --- | --- | --- | --- | --- | --- | --- | --- | --- | --- |
| SL1 | Two-phase + | Two-phase - |  | SL2 | Two-phase + | Two-phase - |  | SL3 | Two-phase + | Two-phase - |
| BMFS + | 2 | 10 |  | BMFS + | 23 | 10 |  | BMFS + | 11 | 13 |
| BMFS - | 7 | 23 |  | BMFS - | 4 | 5 |  | BMFS - | 3 | 15 |
| OR (CI) | 1.4 (0.54, 3.8) | |  | OR (CI) | 2.5 (0.78, 8.0) | |  | OR (CI) | 4.3 (1.2, 15) | |
| *p*-value | 0.481 |  |  | *p*-value | 0.118 | |  | *p*-value | 0.013 |  |
| Comparison of PV detection in BMFS-2 and single BMFS samples and two-phase samples | | | | | | | | | | |
| SL1 | Two-phase + | Two-phase - |  | SL2 | Two-phase + | Two-phase - |  | SL3 | Two-phase + | Two-phase - |
| BMFS + | 2 | 7 |  | BMFS + | 24 | 10 |  | BMFS + | 11 | 11 |
| BMFS - | 7 | 26 |  | BMFS - | 3 | 5 |  | BMFS - | 3 | 17 |
| OR (CI) | 1.0 (0.35, 2.9) | |  | OR (CI) | 3.3 (0.92, 12) | |  | OR (CI) | 3.7 (1.0, 13) | |
| *p*-value | 1.0 |  |  | *p*-value | 0.057 | |  | *p*-value | 0.035 |  |

PV, poliovirus; BMFS, bag-mediated filtration system; WHO algorithm, virus isolation on L20B and RD (human rhabdomyosarcoma) cells followed by ITD (intratypic differentiation); BMFS-1, first collected BMFS sample of the two sequentially collected BMFS samples; BMFS-2, second collected BMFS sample of the two sequentially collected BMFS samples; SL1, Sabin-like PV type 1; SL2, Sabin-like PV type 2; SL3, Sabin-like PV type 3; nd, not determined; OR, Odds ratio; CI, 95% confidence intervals; *p*-value, calculated by the McNemar mid-p test

# **References**

Adams, M. H. (1959). *Bacteriophages*. New York, NY: Interscience Publishers, Inc.

Alexander, J. J., Bey, E. M., Geddes, E. W., & Lecatsas, G. (1976). Establishment of a continuously growing cell line from primary carcinoma of the liver. *South African Medical Journal = Suid-Afrikaanse Tydskrif Vir Geneeskunde*, *50*(54), 2124–2128.

Dahling, D. R., Berg, G., & Berman, D. (1974). BGM, a continuous cell line more sensitive than primary rhesus and African green kidney cells for the recovery of viruses from water. *Health Laboratory Science*, *11*(4), 275–282.

Pipkin, P. A., Wood, D. J., Racaniello, V. R., & Minor, P. D. (1993). Characterisation of L cells expressing the human poliovirus receptor for the specific detection of polioviruses in vitro. *Journal of Virological Methods*, *41*(3), 333–340.

Wood, D. J., & Hull, B. (1999). L20B cells simplify culture of polioviruses from clinical samples. *Journal of Medical Virology*, *58*(2), 188–192.
